# Polio Legacy in Action: Using the Polio Eradication Infrastructure for Measles Elimination in Nigeria—The National Stop Transmission of Polio Program

**DOI:** 10.1093/infdis/jix014

**Published:** 2017-07-01

**Authors:** Charles A. Michael, Ndadilnasiya Waziri, Rajni Gunnala, Oladayo Biya, Katrina Kretsinger, Eric Wiesen, James L. Goodson, Lisa Esapa, Saheed Gidado, Belinda Uba, Patrick Nguku, Stephen Cochi

**Affiliations:** 1 National Stop Transmission of Polio (NSTOP) and; 2 Nigeria Field Epidemiology and Laboratory Training Program (NFELTP), Africa Field Epidemiology Network, Asokoro, Abuja, Nigeria;; 3 Global Immunization Division, Centers for Disease Control and Prevention, Atlanta, Georgia

**Keywords:** Nigeria, polio, measles, legacy.

## Abstract

From 2012 to date, Nigeria has been the focus of intensified polio eradication efforts. Large investments made by multiple partner organizations and the federal Ministry of Health to support strategies and resources, including personnel, for increasing vaccination coverage and improved performance monitoring paid off, as the number of wild poliovirus (WPV) cases detected in Nigeria were reduced significantly, from 122 in 2012 to 6 in 2014. No WPV cases were detected in Nigeria in 2015 and as at March 2017, only 4 WPV cases had been detected. Given the momentum gained toward polio eradication, these resources seem well positioned to help advance other priority health agendas in Nigeria, particularly the control of vaccine-preventable diseases, such as measles. Despite implementation of mass measles vaccination campaigns, measles outbreaks continue to occur regularly in Nigeria, leading to high morbidity and mortality rates for children <5 years of age. The National Stop Transmission of Polio (NSTOP) program was collaboratively established in 2012 to create a network of staff working at national, state, and district levels in areas deemed high risk for vaccine-preventable disease outbreaks. As an example of how the polio legacy can create long-lasting improvements to public health beyond polio, the Centers for Disease Control and Prevention will transition >180 NSTOP officers to provide technical experience to improve measles surveillance, routine vaccination coverage, and outbreak investigation and response in high-risk areas.

In 1988, the Global Polio Eradication Initiative (GPEI) was established to achieve a polio-free world [1]. By 2005, the annual incidence of polio had decreased by >99%, compared with 1988, and wild poliovirus (WPV) elimination was achieved in all countries except Afghanistan, Pakistan, India, and Nigeria [1–2]. However, persistent endemic WPV transmission in these countries led to recurring outbreaks in polio-free countries [3–4]. During 2002–2011, WPV importations primarily from Nigeria led to reestablished WPV transmission and outbreaks in 39 previously polio-free countries [5]. In 2012, the World Health Assembly declared the completion of polio eradication a programmatic emergency.

Nigeria became the focus of intensified efforts by the GPEI partners and the Nigerian government during 2012–2015. In 2012, the Nigerian National Primary Health Care Development Agency, the Nigerian Field Epidemiology and Laboratory Training Program (NFELTP), and the US Centers for Disease Control and Prevention (CDC) established the Nigeria National Stop Transmission of Polio (NSTOP) program. The Nigeria NSTOP design was based on the Stop Transmission of Polio (STOP) program, a key component of GPEI, which was developed and initiated by the CDC with the World Health Organization in 1999 to mobilize additional human resources and technical assistance for countries affected by WPV transmission [6]. Similarly, the Nigeria NSTOP program has created a network of trained staff working at national, state, and district levels in areas deemed high risk for polio transmission because of low vaccination coverage [7].

After the large investments in resources and personnel made by multiple partner organizations and the Nigeria federal Ministry of Health to implement fully the eradication strategies, great strides were made. Confirmed WPV cases decreased from 122 polio cases in 2012 to 6 in 2014, and from July 2014 through September 2016 only 3 cases of polio were detected [8, 9]. The success made toward achieving a polio-free Africa since 2012 provided important momentum for the GPEI and demonstrated the impact that a well-funded eradication program can bring. The fourth goal of the polio endgame strategic plan is to plan polio’s legacy. Moving toward this goal, the polio investments and resources in Nigeria are now being transitioned to advance other priority health agendas [10] in Nigeria, particularly the control and elimination of VPDs , such as measles, in addition to completing the eradication of all poliovirus transmission.

In 2012, the Global Vaccine Action Plan approved by the World Health Assembly set a goal for measles and rubella elimination in at least 5 of the 6 WHO regions by 2020 [11]. The African Region similarly established a goal for measles elimination by 2020 using strategies that include strengthening routine immunization (RI), providing a second opportunity for measles immunization through implementation of periodic high-quality measles campaigns, enhancing epidemiologic surveillance, and improving case management. However, measles outbreaks continue to occur in the region, with an estimated 48000 measles deaths in 2014 [12].

In Africa, Nigeria accounts for the largest number of reported measles cases by country and harbors a persistent measles virus reservoir in the region. To facilitate national measles elimination efforts in Nigeria, the NSTOP funds provided by the CDC can support NSTOP officers in providing technical experience and capacity building to improve measles surveillance, measles vaccination coverage, and outbreak investigation and response in high-risk areas. Using the established NSTOP training program and pool of experienced staff to address measles elimination is a prime example of how the polio legacy, assets, and infrastructure can create long-lasting improvements to public health. To explore and document the full potential of the NSTOP program to enhance efforts to eliminate measles, we conducted an NSTOP legacy planning exercise with a focus on measles elimination, including mapping the complete resources and assets of the NSTOP program in Nigeria, and we reviewed annual reported measles cases and estimated measles vaccination coverage.

## METHODS

The CDC and the NSTOP program, as major stakeholders in the Nigeria polio infrastructure, conducted a transition planning exercise following the methods outlined in the polio legacy planning guidelines for preparing a transition plan [13, 14]. A focus of the exercise was how to transition the NSTOP assets to address measles elimination in Nigeria. The process used for selected steps in the legacy planning exercise is outlined below.

### Mapping NSTOP Polio Program Resources

NSTOP polio program resources at the country level, comprising both “assets” and “functions,” were mapped to ascertain the size, structure, location, activities and their suitability for measles intervention. Assets mapped included all human resources, physical infrastructure, and financing structures established by the program across the NSTOP-supported states in Nigeria. The asset mapping was conducted by cataloguing all staff and other resources with the assistance of the NSTOP program administrator. NSTOP program functions are the systems, processes, and activities that the program carries out [13]. The function mapping was conducted by reviewing the terms of reference of all NSTOP staff members to ascertain the functions they perform.

### Documenting Lessons Learned

In 2015, NSTOP program undertook an internal assessment. The main objective of the assessment was to assess the program’s contribution to the implementation of the polio eradication and RI programs in Nigeria. The assessment included in-depth interviews with key stakeholders, surveys, focus group discussions (FGDs), and secondary data analyses. Interviews and surveys were done using a pretested interview guide and semistructured questionnaire, respectively, to collect data on activities of NSTOP officers, the impact of NSTOP program support, and likely effects of withdrawal of NSTOP officers. All national-level and state-level officers in all 12 NSTOP-supported states and the Federal Capital Territory who had interacted with the NSTOP program at the management level were interviewed. In addition, stratified random sampling proportional to the number of local government areas (LGAs) in each state was used to select 38 LGAs from 184 NSTOP-supported LGAs, and simple random sampling was used to select 3 health facilities in each LGA. Security-compromised LGAs in Borno, Yobe, and Adamawa states were excluded. Stratification was based on the phases by which NSTOP activities were rolled out in the LGAs. 

NSTOP activities commenced in 3 phases: phase 1 in April 2013, phase 2 in July 2013, and phase 3 in June 2014. Of the 38 selected LGAs, 10 are in phase 1 and 14 each in phases 2 and 3. The sample of partner and government officers included 17 national-level, 78 state-level, 111 local LGA–level, and 181 health facility–level officers with a response rate >90% across all levels. FGDs were conducted with NSTOP LGA officers (NSLOs), using a guide to collect data on NSLOs experience with supporting immunization activities in the LGAs. Seven FGDs with 8–10 NSLOs per FGD were conducted in 7 purposively selected NSTOP-operational states. Survey data was cleaned and analyzed using SAS 9.3 software (SAS Institute, 2013). We determined frequencies and proportions of variables. Qualitative data were transcribed and read multiple times for proper understanding. Coding and thematic analysis were done using ATLAS.ti 7 (ATLAS.ti Scientific Software Development GmbH), and program records were analyzed using Microsoft Excel (Version 15, 2013). In addition, the NSTOP program reviewed reports of its technical support for the 2015–2016 nationwide measles supplementary immunization activities (SIAs) to document lessons learned and draw from such lessons to develop the NSTOP polio-measles transition framework.

### Linking NSTOP Polio Infrastructure to National Health Priorities

The Nigeria Expanded Programme on Immunization country multiyear plan for 2016–2020 was reviewed to identify national health priorities. Further literature review was undertaken to identify regional health priorities to which Nigeria is a signatory.

### Mapping of Measles Cases

Measles cases are reported in Nigeria through the measles case-based surveillance system adopted in 2005 and the Integrated Disease Surveillance and Response system. The LGA disease surveillance and notification officer receives immediate notification on all suspected measles cases reporting at the health facility. This officer then investigates the cases using standardized forms (Integrated Disease Surveillance and Response 001A and 1B) and also collects blood specimens for laboratory testing. The data obtained from health facilities are sent to the LGA, which then compiles and sends the data to the epidemiology unit of the state Ministry of Health for onward transmission to the epidemiology division of the federal Ministry of Health and WHO country office. The federal Ministry of Health publishes a weekly epidemiology report using these data. Weekly epidemiology reports from the federal Ministry of Health (from week 1 in 2013 to week 32 in 2016) and accelerated disease control feedback from WHO country office (January–August 2016) were reviewed. Reported measles cases were aggregated and mapped by state to ascertain geographic distribution of measles burden in relation to the mapped NSTOP resources, processes, and experiences.

### Determining Appropriate Transition Strategy

A 1-day planning meeting was held to determine the specific strategies for applying the mapped NSTOP polio infrastructures to the Nigeria measles elimination program. Fifteen persons participated in the planning meeting, including 4 representatives of the Nigeria polio team, Global Immunization Division, CDC, Atlanta; 1 Global Immunization Division staff member from the Nigeria Center for Disease Control; 6 NSTOP national-level epidemiologists; 3 NSTOP data managers; and 1 NSTOP senior administrator. Different transition strategies were developed for subsets of NSTOP assets, functions, or lessons learned to address the different aspects of the measles program in Nigeria. The strategies were then aggregated into a business case document.

## RESULTS

### Mapping NSTOP Polio Program Resources

NSTOP assets were found to include people, physical assets, and polio program-specific experiences; NSTOP functions included implementation planning and service delivery; monitoring and supportive supervision, data management, and research; disease surveillance; community engagement, communication, and political advocacy; capacity building; resource mobilization and coordination; policy development, strategic planning and oversight; partnerships and coordination; and management and accountability (Table 1) [13].

**Table 1.** Nigeria NSTOP Program Functions, 2016

Function	Primary Subfunctions	Types of Activities	Personnel Involved
Implementation planning and service delivery	Provide support for priority states in polio eradication and SIA; strengthen RI; support nonpolio VPD and non-VPD health initiatives; MSTs	SIA planning and implementation, outbreak investigation and response, logistical support; supportive supervisory visits for fixed and outreach sessions; support conduct of measles SIA, support activities to reduce malaria burden; deploy MSTs to support SIAs especially in hard-to-reach areas	Senior field coordinator (1), state field coordinator (17), NSLOs (184), RI field coordinator (1); (203 total)
Monitoring, data management, and research	Monitoring and evaluation; technical support for development of a high-quality system for monitoring national RI; operations research; data analysis and reporting	Monitor progress of NSTOP program activities; provide leadership on the use of DHIS2 dashboard; conduct operational research studies; support use of data for decision making	NSLOs (184), DHIS field coordinator (1), data manager (5), DHIS implementation officer (3), data technical officer (4), monitoring and evaluation officer (1); (198 total)
Disease surveillance	Strengthening AFP and other VPD surveillance	Support activities to strengthen AFP and other VPD surveillance	NSLOs (184), state field coordinators (17), surveillance field coordinator (1); (202 total)
Community engagement, communications and political advocacy	Build relationships with local community leaders, partner organizations, and federal Ministry of Health; demand creation for RI and healthcare services; increase government support for program objectives	Enlist community leaders and conduct community dialogue to improve RI acceptance, advocate for government ownership	NSLOs (184), state field coordinators (17), surveillance field coordinator (1); (202 total)
Capacity building	Training	Provide training in 9 RI thematic areas and conduct posttraining field assignments	Senior field coordinator (1), state field coordinators (17), NSLOs (184), RI field coordinator (1), training field coordinator (1), data technical officers (4), DHIS field coordinator (1), DHIS implementation officers (3); (total 212)
Resource mobilization and donor coordination	Resource mobilization	Advocate to government and partners for sustainable funding for NSTOP activities	National coordinator (1), deputy national coordinator (1), NSTOP administrator (1); (3 total)
Policy development, strategic planning, and oversight	Participate in national, state, and LGA level meetings on immunization policy and strategic plans; provide data and information at the national and state levels for decision making	Participate in task force and working group meetings at all levels	State field coordinators (17), NSLOs (184); (201 total)
Partnerships and coordination	Work closely with other PEI partners to make evidence-based decisions to improve campaign planning and implementation	Coordinate with government and partner organizations to achieve polio eradication objectives, support control of other outbreaks (eg, Ebola)	Senior field coordinator (1), state field coordinators (17), RI field coordinator (1), national coordinator (1), deputy national coordinator (1), training field coordinator (1), DHIS field coordinator (1), surveillance coordinator (1), technical support to emergency operation center (1); (25 total)
Management and accountability	General management; general administration	Prepare and update annual work plans, schedule quarterly review meetings.	National coordinator (1), deputy national coordinator (1), NSTOP administrator (1), program administration team (14); (17 total)

Abbreviations: AFP, acute flaccid paralysis; DHIS, District Health Information System; LGA, local government area; MSTs, management support teams; NSLOs, NSTOP LGA officers; NSTOP, National Stop Transmission of Polio; PEI, polio eradication initiative; RI, routine immunization; SIA, supplementary immunization activity; VPD, vaccine-preventable disease.

**Figure 1.**
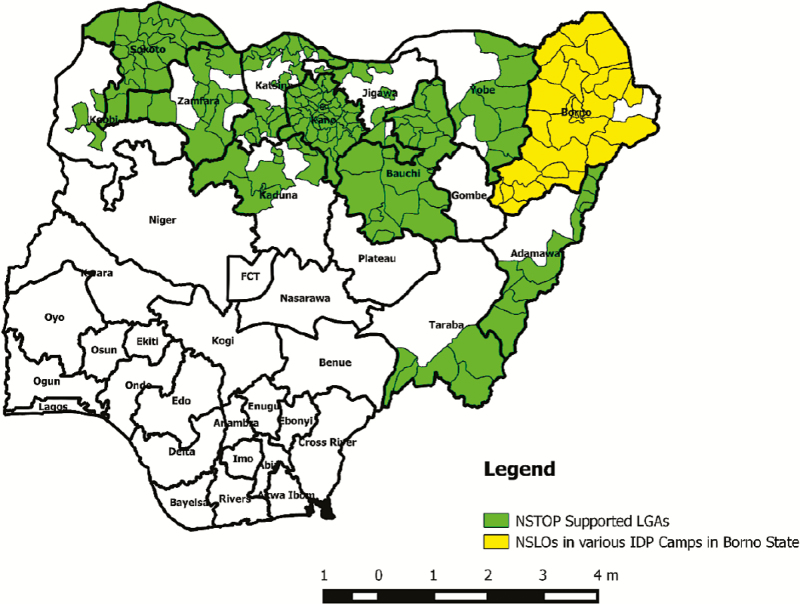
Map of Nigeria showing distribution of Nigeria National Stop Transmission of Polio (NSTOP)–supported local government areas (LGAs), including LGAs supported in security-compromised Borno state, 2013–2016. Abbreviations: FCT, Federal Capital Territory; IDP, internally displaced person; NSLOs, NSTOP LGA officers.

The NSTOP program has 285 officers at LGA, state, and national levels who can support measles elimination activities in Nigeria. NSTOP has 1 NSLO in each of 184 LGAs in northern Nigeria (Figure 1). They support the LGA to build staff capacity, update RI and campaign microplans, provide supportive supervision to health facilities, improve campaign quality and surveillance, and respond to outbreaks.

The NSTOP program has also trained >400 NFELTP residents in vaccine-preventable disease (VPD) programs and frequently uses this pool of trained personnel for field exercises such as management support for SIAs. These serve as a readily available pool of trained and experienced personnel who can be mobilized to improve measles surveillance, data quality, vaccine coverage, and outbreak response.

### Documenting Lessons Learned

The majority of respondents in the NSTOP assessment, excluding NSTOP staff, reported that the NSTOP program has had a positive effect on the polio eradication efforts and the overall immunization program in Nigeria. There was consensus that NSTOP officers carried out immunization activities among Fulani (a nomadic group) and other hard-to-reach communities. The majority of respondents from health facilities reported that NSTOP trainings had improved their knowledge and skills on RI and supported RI outreach sessions to underserved population and that NSTOP officers regularly supervise their RI sessions. At the LGA and state levels, the majority reported that NSTOP officers performed activities such as support for polio campaigns, supportive supervision for RI, support for training on RI, and facilitation of quarterly RI microplan update and that they performed these activities as often as planned. The majority also reported that there would be a negative effect on immunization activities at the LGA and state levels if NSTOP officers were withdrawn. There was also a majority opinion from LGAs and states that the NSTOP program has insufficient staff. The top drivers for program expenditures overall were capacity building for RI strengthening, RI data quality improvement, and deployment of management support teams for polio campaigns.

### Best Practices

The model of the NSTOP program is to build capacity of the entire immunization team at the LGA level. Therefore, it developed a series of 9 thematic modular trainings and provided training to NSTOP as well as government and partner staff at the LGA and health facility levels (the 9 RI thematic modules included introduction to RI, reach-every-ward microplanning, vaccine and cold chain management, RI service delivery and supportive supervision, campaign management, data management, demand creation, VPD surveillance, and monitoring and evaluation). The modules included a practical exercise at the end of each training, which was useful in building the capacity of trained participants and in gathering additional data for overall RI improvement at the LGA. The NSTOP program focused heavily on ensuring that traditionally historically unreached communities, such as nomadic groups and scattered settlements, were indeed captured in microplans and reached by polio campaigns and surveillance activities. It also conducted useful rapid field data collection, including enumeration of nomadic children, RI surveys, and qualitative surveys of the reasons for missed children and vaccine refusers. The NSTOP model involves close mentorship and support and partnership with the NFELTP.

The NSLOs have successfully integrated themselves with the LGA immunization team and participate in decision making to improve overall program performance. To improve vaccine coverage, NSLOs conduct an average of 12 supportive supervisory visits per month in health facilities serving mainly underserved communities. They also use this opportunity to conduct active case search for VPDs and provide on-the-job training as needed. During outbreaks, NSLOs are deployed rapidly as part of the outbreak response team. In 12 states, NSTOP state field coordinators support the state immunization team to make effective management decisions and coordinate LGA-level activities. They are active members of the relevant state emergency operation centers and support activities at the LGA as needed.

**Figure 2.**
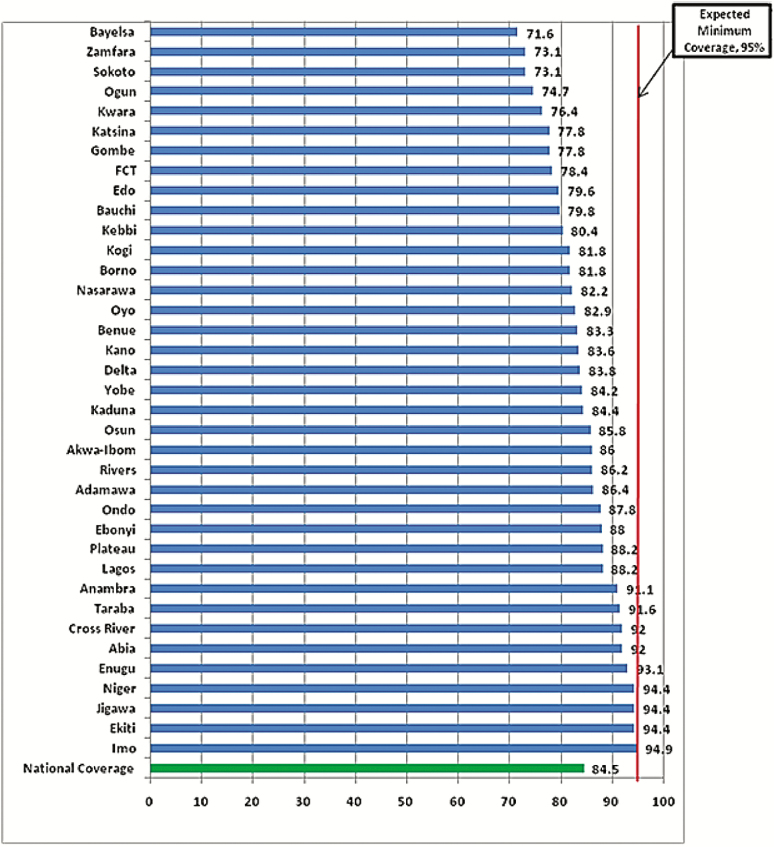
Measles coverage by states in the 2015–2016 supplementary immunization activity in Nigeria (n = 16650). Source: 2016 measures Hanovia Medical Limited measles vaccination coverage survey report. Abbreviation: FCT, Federal Capital Territory.

The NSTOP program supported the 2015–2016 measles SIA across all phases of implementation. The support included facilitation at trainings, development and monitoring of a measles SIA dashboard, supportive supervision, and on-the-job training for measles vaccination teams. In addition, the program supported the independent postcampaign coverage survey undertaken to assess the quality and coverage of the measles SIA. Contributions from NSTOP, federal, state, and LGA actors as well as other partners led to an increase in national measles SIA coverage from 74% in 2013 to 84% (Figure 2) in 2016.

### Link with National Priorities

Measles is one of the national health priorities in Nigeria. The Nigeria Expanded Programme on Immunization country multiyear plan for 2016–2020 includes a goal of reducing measles morbidity and mortality by >90% by 2020. In addition, Nigeria is a signatory to an World Health Organization African Regional Office (AFRO) regional measles elimination target date of 2020 that was passed by the African Regional Committee in September 2011 [15].

### Measles Epidemiology

**Figure 3.**
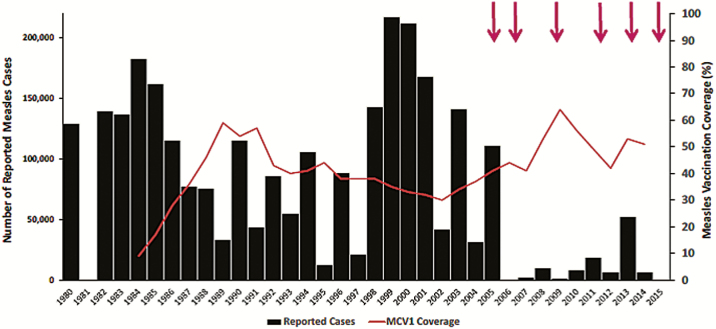
Reported number of measles cases and estimated coverage with the first dose of measles-containing vaccine (MCV1), Nigeria, 1980–2015 [16, 17]. Arrows indicate phased national measles vaccination campaigns, according to WHO data (http://ww.who.int/immunization/monitoring_surveillance/data/en).

Measles cases decreased considerably after the implementation of a catch-up immunization campaign in 2005. However, there was a large resurgence of measles in 2013 with over 50000 cases recorded [18] (Figure 3). Although follow-up campaigns have been conducted regularly since 2005, including the most recent campaign in 2015–2016, 17378 measles cases were reported between January and May 2016, compared with 9741 for the same time period in 2015 [19]. Routine measles vaccination coverage remains low in Nigeria and 62% of confirmed (laboratory confirmed, epidemiologically linked, clinically compatible) measles cases during January–March 2016 were not vaccinated against measles [20]. The majority of cases in 2016 occurred in 19 northern states of Nigeria. NSTOP resources are present in 12 of these 19 Northern states and can be harnessed to drive the control of measles and other VPD.

### Transition Strategy

An NSTOP transition strategy was developed with a focus on measles control. The main components of the strategy include enhancing measles surveillance, increasing measles vaccination coverage, and improving measles outbreak response activities. The scope for the NSTOP support is the 184 LGAs with an NSTOP officer as well as ad hoc support to other areas as needed. The main activities include conducting a baseline assessment to gauge current knowledge and practices related to measles control, conducting a measles-specific thematic training for the LGA team in the 184 NSTOP-supported LGAs, conducting field activities to improve measles control among high risk and hard-to-reach communities, and participating in measles outbreak response activities as needed. The strategy will be implemented in 2017. Already, a national NSTOP measles focal person has been appointed to provide central-level coordination and field-based support for program activities at state and LGA levels.

## DISCUSSION

With the decline in WPV cases to an all-time low during 2014–2016, Nigeria has demonstrated very powerfully the impact that can be gained by well-placed investments in health. These investments have the potential to make a major impact on other priority health issues in Nigeria after the eradication of poliomyelitis. Measles is among the 5 preventable infectious diseases contributing to >70% of the estimated 1 million deaths in children <5 years old in Nigeria [21]. Thus, measles control and elimination is a major national health priority in Nigeria and could be considered the next major focus in VPD prevention in Nigeria. Given the size and mobility of the Nigerian population, controlling measles in Nigeria is critical to achieving regional and global measles goals as well.

This report demonstrates the potential for the polio infrastructure in Nigeria to be transitioned effectively to other public health priorities, such as measles elimination. The technical capacity amassed to fight polio in Nigeria is enormous. The NSTOP program is just one small piece of the total polio infrastructure in Nigeria, and if the entire might of the polio program in Nigeria were applied to measles control and RI strengthening, an immense impact similar to that of polio eradication could be expected.

Nevertheless, major challenges exist in transitioning these resources. Creating a common vision of the future of the polio infrastructure in Nigeria requires collaboration between multiple units within the government of Nigeria as well as multiple partners and donor agencies. Securing funding for continuing of some portion of the surge in staff that was created to stop polio will be challenging as donors grapple with competing priorities and limited resources. Ideally, many of the polio functions would be taken over by government, but in the context of the current economic downturn in Nigeria this may be very difficult to achieve. Finding the right structure and placement for the polio resources in a postpolio era is also a complex issue, because the structure has largely existed outside the permanent government agencies.

The current study is subject to several limitations. First, the measles case data are likely to be an underestimate of the true extent of measles transmission. This is due in part to the limited active field surveillance and the tendency for patients with mild measles to stay at home. Moreover, the data used to document the lessons learned regarding the NSTOP program covered only 38 LGAs and did not include security-compromised LGAs. Finally, the proposed support for measles control has not yet been tested, and we do not yet have evidence regarding the effectiveness of this approach.

However, as Nigeria approaches full interruption of poliomyelitis, it is envisioned that this approach of transitioning polio resources to address measles control will lead to real and lasting reductions in measles morbidity and mortality in Nigeria. Although there is a great opportunity now to transition polio resources to address other priority public health problems, in the absence of clear and unified plans, there is a real risk that these resources will be withdrawn in the near future, leaving Nigeria with a greatly diminished capacity to address its public health priorities. We hope this effort will assist in avoiding such an outcome.

## Notes

***Financial support.*** This work was supported by the US CDC.

***Supplement sponsorship.*** This work is part of a supplement coordinated by the Task Force for Global Health with funding provided by The Bill and Melinda Gates Foundation and the Centers for Disease Control and Prevention.

***Potential conflicts of interest.*** All authors: No reported conflicts. All authors have submitted the ICMJE Form for Disclosure of Potential Conflicts of Interest. Conflicts that the editors consider relevant to the content of the manuscript have been disclosed.
